# Epigenetics of Notch1 regulation in pulmonary microvascular rarefaction following extrauterine growth restriction

**DOI:** 10.1186/s12931-015-0226-2

**Published:** 2015-06-04

**Authors:** Li-Li Tang, Li-Yan Zhang, Lin-Jiang Lao, Qiong-Yao Hu, Wei-Zhong Gu, Lin-Chen Fu, Li-Zhong Du

**Affiliations:** Department of Neonatology, The Children’s Hospital, Zhejiang University School of Medicine, Hangzhou, 310051 People’s Republic of China; Department of Neonatology, The Children’s Hospital of Fuzhou, Fujian Medical University, Fuzhou, 350004 People’s Republic of China; Department of Pathology, The Children’s Hospital, Zhejiang University School of Medicine, Hangzhou, 310051 People’s Republic of China

**Keywords:** Extrauterine growth restriction, Pulmonary endothelium, Notch1, Epigenetics

## Abstract

**Background:**

Extrauterine growth restriction (EUGR) plays an important role in the developmental origin of adult cardiovascular diseases. In an EUGR rat model, we reported an elevated pulmonary arterial pressure in adults and genome-wide epigenetic modifications in pulmonary vascular endothelial cells (PVECs). However, the underlying mechanism of the early nutritional insult that results in pulmonary vascular consequences later in life remains unclear.

**Methods:**

A rat model was used to investigate the physiological and structural effect of EUGR on early pulmonary vasculature by evaluating right ventricular systolic pressure and pulmonary vascular density in male rats. Epigenetic modifications of the Notch1 gene in PVECs were evaluated.

**Results:**

EUGR decreased pulmonary vascular density with no significant impact on right ventricular systolic pressure at 3 weeks. Decreased transcription of Notch1 was observed both at 3 and 9 weeks, in association with decreased downstream target gene, *Hes-1*. Chromatin immunoprecipitation and bisulfite sequencing were performed to analyze the epigenetic modifications of the Notch1 gene promoter in PVECs. EUGR caused a significantly increased H3K27me3 in the proximal Notch1 gene promoter, and increased methylation of single CpG sites in the distal Notch1 gene promoter, both at 3 and 9 weeks.

**Conclusions:**

We conclude that EUGR results in decreased pulmonary vascular growth in association with decreased Notch1 in PVECs. This may be mediated by increased CpG methylation and H3K27me3 in the Notch1 gene promoter region.

**Electronic supplementary material:**

The online version of this article (doi:10.1186/s12931-015-0226-2) contains supplementary material, which is available to authorized users.

## Background

Increased risk of adult-onset cardiovascular dysfunction may originate from nutritional disturbances during critical windows of development [1], including prenatal [2, 3] and early postnatal period [4–6]. Increasing evidence also supports the early origins of later onset pulmonary vascular disease. Infants with a transient perinatal insult may develop pulmonary arterial hypertension early in postnatal life [7], or be predisposed to an increased risk of developing this disorder later in life [8]. In animal studies, we recently addressed a strong association between malnutrition during different critical windows and impaired pulmonary vascular function in adulthood [9–11]. Intrauterine growth restriction (IUGR) resulted in aggravated hypoxic pulmonary hypertension and vascular remodeling [10], whereas extrauterine growth restriction (EUGR) rats developed elevated pulmonary arterial pressure (PAP) in the absence of a second-hit [11]. Although we and other study [12] provide the evidence about developmental plasticity of pulmonary vasculature, the precise mechanism is still unknown.

In searching the mechanisms underlying these adult-onset phenotypes, growing data support the hypothesis that physiological adaptations in response to early nutritional deprivation may induce permanent alterations of structure in the developing cardiovascular system. Microvascular rarefaction, a predictor of adverse cardiovascular prognosis, has been observed in the very early stages of systemic hypertension [13–15] as well as in pulmonary arterial hypertension [16]. A strong association between vascular rarefaction and nutritional programming of elevated BP has also been addressed in adult animal models [17–19]. However, whether EUGR exerts a negative impact on early pulmonary vasculature in the development of pulmonary arterial hypertension is presently unknown.

Dysregulation of genes involved in impaired angiogenesis may result in microvascular rarefaction. Notch1 signaling, predominantly in the endothelium, plays a critical role in the development of the cardiovascular system [20]. It has been validated as an essential contributor to prenatal and postnatal angiogenesis [21, 22] by activating downstream genes, such as transcription factor hairy and enhancer of split-1(*Hes-1*) [23]. Notch1 is also identifiable in larger vessels in the embryonic lung, finer alveolar vascular networks in later fetus, and postnatal period [22, 24], suggesting an important role in pulmonary vascular development.

Epigenetic regulation, including histone modification and DNA methylation, plays a major part in the developmental origins of adult diseases [25, 26]. Our recent studies revealed both IUGR and EUGR induced histone modifications in the vascular tone regulators, endothelin-1 (ET-1) and endothelial nitric oxide synthase (eNOS) [10, 11]. In addition, expression of key factors, peroxisome proliferator-activated receptor gamma (PPARγ) and insulin-like growth factor-1 (IGF-1), were affected by DNA methylation and histone modification following EUGR [11]. In line with other evidence [27], we postulate that early nutritional experience has a wide impact on the epigenetic modification of genes involved in normal lung development and morphogenesis. An increasing number of studies have documented the epigenetic dysregulation of Notch1 in different tissues and cells [28–30], including H3K9/14ace, H3K27me3 changes and DNA methylation at the gene promoter. However, the epigenetic regulation of Notch1 in pulmonary endothelium remains unclear.

In the present study, we sought to determine whether EUGR affects the pulmonary vasculature, which may be a contributor to pulmonary vascular dysfunction in later life. We further hypothesized that dysregulation of Notch1in pulmonary endothelium is involved in abnormal alteration of vascular structure through epigenetic adaptation secondary to EUGR.

## Methods

### Extrauterine growth restriction rat model

The EUGR rat model was established as described in our previous study [11]. Briefly, pregnant Sprague–Dawley rats were purchased from Zhejiang University Laboratory Animal Center in Hangzhou, China, and maintained on standard chow diets throughout gestation. Pups were weighed within 24 h of birth and randomly assigned to either a control litter, consisting of 8–10 pups, or a larger litter consisting of 18–20 pups, both with a 1:1 male-to-female ratio. Pups were weaned on postnatal day 21, and pups in the large litter whose weight was below the 10th percentile of age-matched controls were considered as EUGR [31]. Male rats were housed five per cage and fed *ad libitum*. All procedures and experiments were approved by The Animal Care and Use Committee of Zhejiang University, China. Body weights were recorded at 3, 6, and 9 weeks.

### Pulmonary arterial pressure measurements

Right ventricular systolic pressure (RVSP) of rats at 3 weeks of age was evaluated as described previously (we use RVSP to represent systolic pulmonary pressure in 3-week-old animals in this study because for this study group, it is not possible to insert a line into the pulmonary artery at 3 weeks of age) [9]. Briefly, rats were anesthetized with chloral hydrate (100–400 mg/kg, intraperitoneally) and connected to a ventilator. Body temperature was maintained at 38 °C. A PE21 catheter was inserted into the right ventricle via the right jugular vein, which was connected to a pressure transducer. RVSP was recorded using a computer data acquisition system (RM6240B/C, Chengdu Instruments, Chengdu, China).

For measurement of mean PAP, rats at 9 weeks of age were anesthetized and placed on a thermo-regulated surgical table. A PE-50 catheter with the angle directed anteriorly was inserted from the right jugular vein through the right heart into the main pulmonary artery. Placement at each stage was confirmed by respective pressure contours. Hemodynamic values were measured and automatically calculated by a physiological data acquisition system (Acknowledge MP150; Biopac System Inc., Goleta, California, USA) as described previously [11].

### Immunohistochemistry and morphometric analysis

Left lungs were isolated from rats and inflated with ice-cold 10 % formalin in phosphate-buffered saline (PBS) and fixed for at least 48 h at 4 °C. Fixed lung tissue was paraffin embedded, sectioned at 4–5 μm, and processed for light microscopic immunohistochemistry. Samples were incubated overnight with a primary antibody against von-Willebrand factor (Dako, Glostrup, Denmark), an endothelium marker, followed by a secondary antibody (HRP polymer) for 30 min at 37 °C. At least five fields of view from different lung sections per animal were evaluated. Pulmonary vascular density was determined by counting the number of positively stained small pulmonary vessels (<50 μm) per high-powered field (× 200). Sections of peripheral lung were examined, and fields with large airways or major vessels were avoided. The measurements based on each field for each animal were averaged to derive the mean number for that animal. The mean for each animal was used for statistical analysis [32].

### Isolation of pulmonary vascular endothelial cells

Rat pulmonary vascular endothelial cells (PVECs) were isolated by magnetic-activated cell sorting (MACS) according to the procedure in our previous study [10]. Rat lungs were perfused with PBS into the trachea following anesthetization and exsanguination via the abdominal aorta. Fresh detached lung tissue was sliced into 1 mm pieces and incubated in a 37 °C water bath with collagenase A. Lung cell suspensions were filtered, centrifuged, and washed in MACS running buffer. The pellets were resuspended in MACS running buffer, and then incubated with PECAM-1 (BD PharMingen, San Diego, California, USA) in a dark place for 15 min at 4 °C. After washing twice, the pellets were resuspended and incubated in anti-PE MicroBeads (Miltenyi Biotec, Bergisch Gladbach, Germany). After washing, the final cell suspension was added to a large column. Cells which were positively labeled with magnetic microbeads and PECAM-1 antibody were selected using the separation unit, quickly frozen in liquid nitrogen, and kept at −80 °C until use.

### Total RNA isolation and quantitative RT-PCR

Total RNA and genomic DNA were simultaneously purified from PVECs kept at −80 °C according to the isolation kit protocol (Omega, Doraville, GA, USA). RNA was reverse transcribed with a reverse transcriptase kit (Takara, Dalian, China). Real-time quantitative PCR was performed by LightCycler 480 Instrument (Roche, USA) following the Takara SYBR-Green (Takara, Dalian, China) protocol. β-Actin was used as an internal control. All primers are shown (see Additional file 1: Table S1).

### ChIP assay and ChIP qPCR

A chromatin immunoprecipitation (ChIP) assay was performed as described previously [11]. Briefly, isolated PVECs were fixed and cross-linked, and then lysed on ice. Lysed extracts were subjected to shearing by sonication and centrifuged to collect the soluble protein-chromatin complex. The soluble chromatin was divided equally and incubated overnight with: anti-acetyl-H3K9 (Millipore 07–352, Billerica, MA, USA), anti-acetyl-H3K14 (Millipore 07-353, Billerica, MA, USA), anti-me3-H3K9 (Millipore 17–625, Billerica, MA, USA), and anti-me3-H3K27 (Millipore 17–622, Billerica, MA, USA). The complexes were precipitated with protein A-agarose beads and incubated with NaCl to reverse DNA and protein crosslink. Finally, DNA fragments were purified with a Qiagen DNA extraction kit (Qiagen, Valencia, CA, USA). Relative quantification of PCR products was based on value differences between the bound and input using the ∆∆Ct method. The primers used for ChIP qPCR are shown elsewhere (see Additional file 1: Table S1).

### Bisulfite sequencing

PVEC genomic DNA was extracted (Omega, Doraville, GA, USA) and, according to the instructions of EpiTect Bisulfite kit (Qiagen, Valencia, CA, USA), 2 μg was treated with sodium bisulfite. Based on the different enrichment peaks detected in the distal Notch1 gene promoter previously [11], two significantly hypermethylated regions within the peak were selected. The primers for bisulfite sequencing (BS) of regions (BS1, BS2) are reported (see Additional file 1: Table S1). At least 10 clones per sample were sequenced to assess the methylation state of the Notch1 gene promoter.

### Statistical analysis

Repeated-measures ANOVA and student’s 2-tailed *t*-test were used for independent samples. Correlation between gene expression and DNA methylation was analyzed using the Pearson test. A *P* value <0.05 was considered statistically significant.

## Results

### Effect of extrauterine growth restriction on body weight and pulmonary arterial pressure

**Fig. 1:**
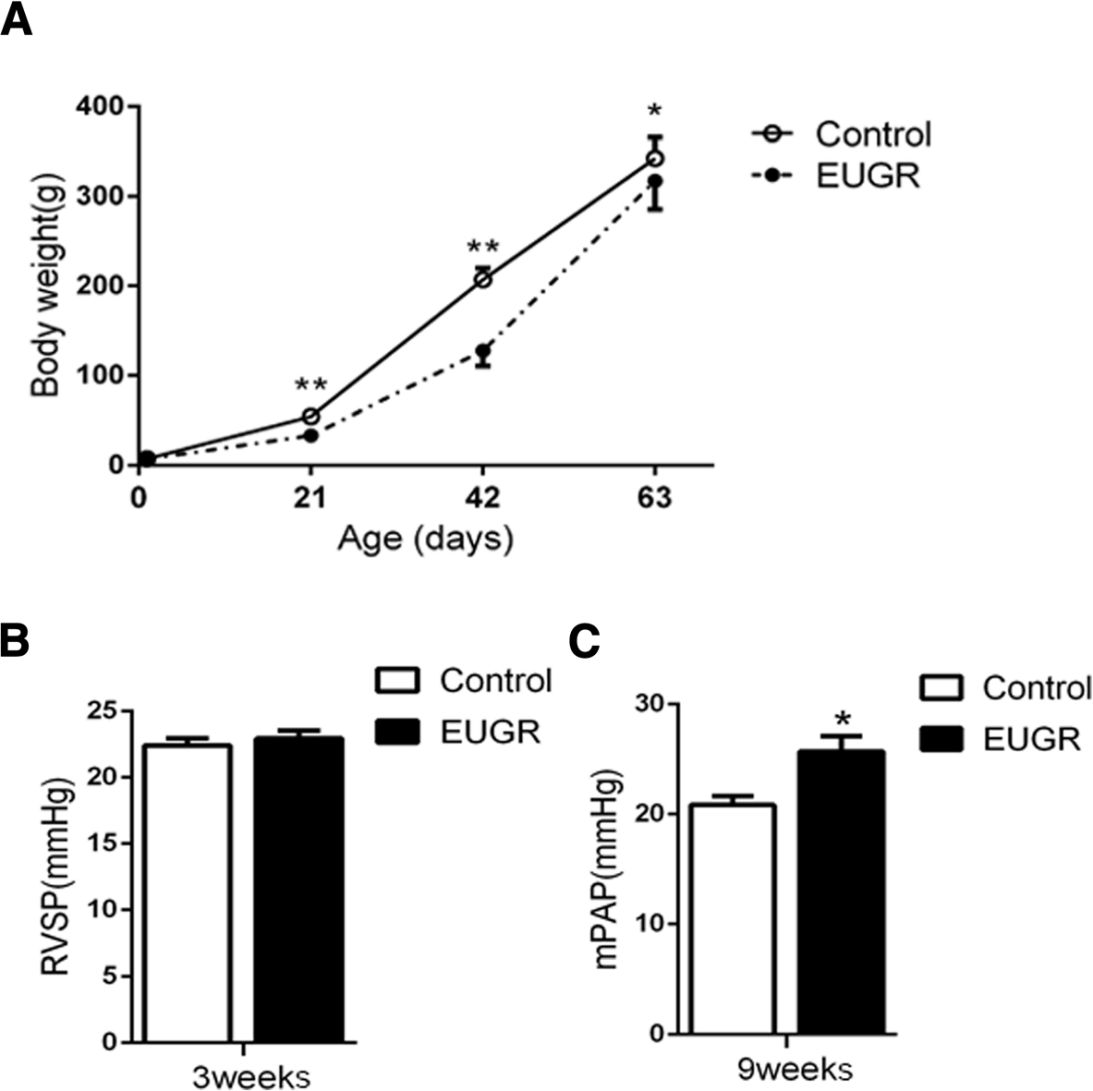
Effect of extrauterine growth restriction (EUGR) on body weight, right ventricular systolic pressure (RVSP), and pulmonary arterial pressure (PAP). **a**: Body weight from birth to 9 weeks of control and EUGR rats. Data are expressed as means ± SEM from 20–25 animals per group at each time point. **b**: RVSP of 3- week-old rats. **c**: mean PAP (mPAP) of 9-week-old rats. Data are expressed as means ± SEM from 5 animals per group. *P < 0.05, **P < 0.01

There was no significant difference between the EUGR and control rats for birth weight. After weaning at 3 weeks, the body weight of EUGR rats was significantly less than that of the control rats (Fig. 1a, *P =* 0.0002). This difference persisted in mature rats. Although weight gain was greater in EUGR rats after weaning, compared with controls, EUGR rats didn’t show a complete catch-up in growth by 9 weeks (Fig. 1a, *P* = 0.016). RVSP of rats at 3 weeks of age, which represents systolic PAP in this study, and PAP of 9 week-old rats were recorded to examine the effect of EUGR on hemodynamics. There were no significant differences between EUGR and control rats for RSVP at 3 weeks (20.92 ± 0.58 mmHg *vs* 20.40 ± 0.55 mmHg, *P =* 0.537, Fig. 1b). Mean PAP was increased in the EUGR compared with the control group at 9 weeks (25.72 ± 1.36 mmHg *vs* 20.83 ± 0.83 mmHg, *P =* 0.012, Fig. 1c), which illustrated the effect of EUGR on PAP in response to mismatched postnatal nutritional challenge, as we reported previously [11].

### Effect of extrauterine growth restriction on pulmonary vascular density

**Fig. 2:**
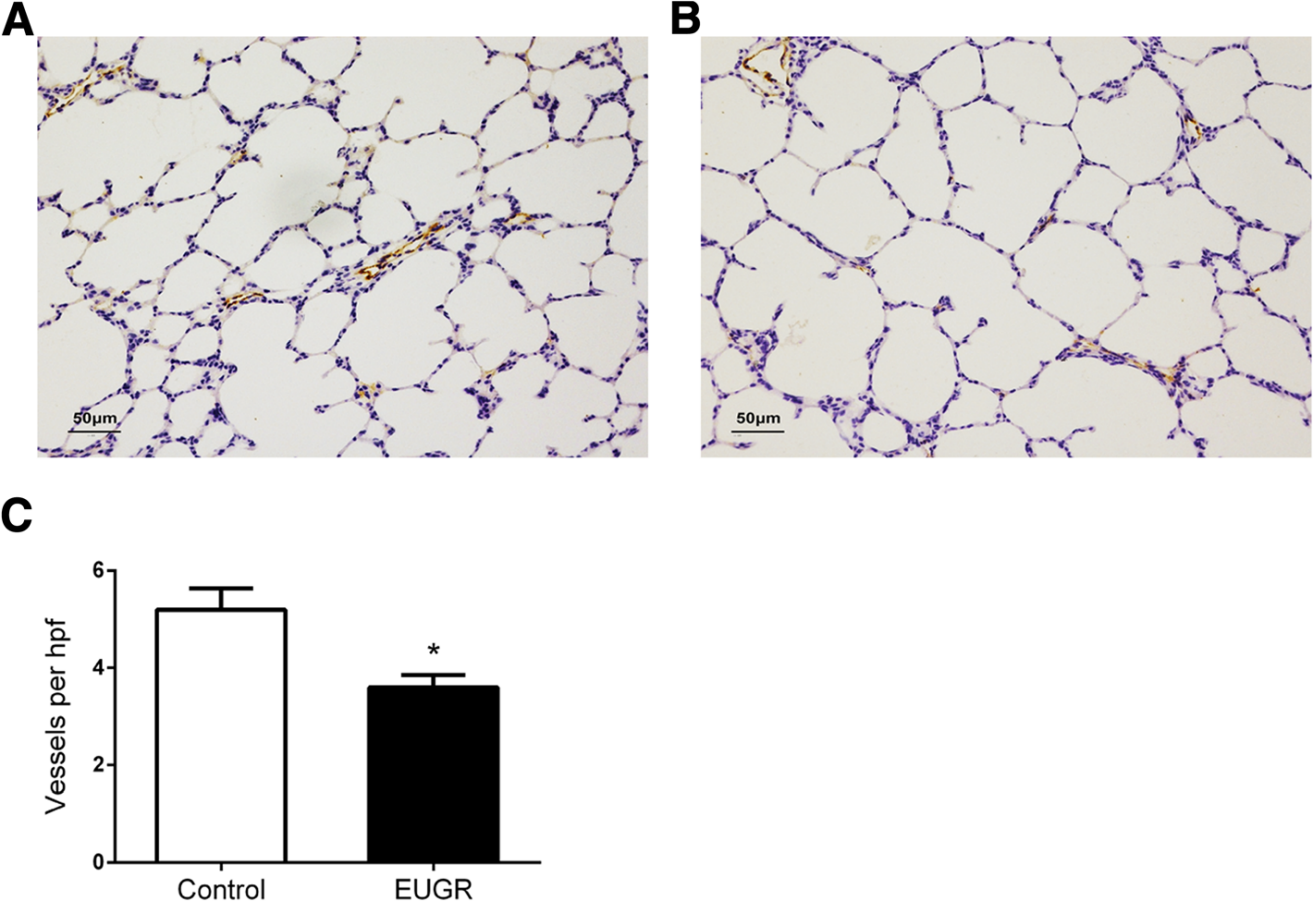
Effects of EUGR on postnatal pulmonary vessel density after weaning. Representative lung histology from control (**a**) and EUGR (**b**) rats at 3 weeks, stained with von Willebrand factor (a marker of endothelium). Micrographs were obtained at the same magnification (200 ×). Internal scale bar (50 μm). **c**: the number of von Willebrand factor-stained pulmonary vessels (diameter <50 μm) per high-power field decreased in EUGR rats. Data are presented as mean ± SEM. (n = 5 rats per group). *P < 0.05

Pulmonary vascular density (<50 μm) was measured by immunohistochemical staining of von-Willebrand Factor in five rats from each group at 3 weeks. The effects of EUGR on postnatal pulmonary vascular density at 3 weeks are shown in Fig. 2. Compared with controls, pulmonary vascular density was decreased in the EUGR rats (Fig. 2c, *P* = 0.013); no between group differences in pulmonary vascular density were found in adulthood (data not shown).

### Effect of extrauterine growth restriction on mRNA level of Notch1 and downstream genes

**Fig. 3:**
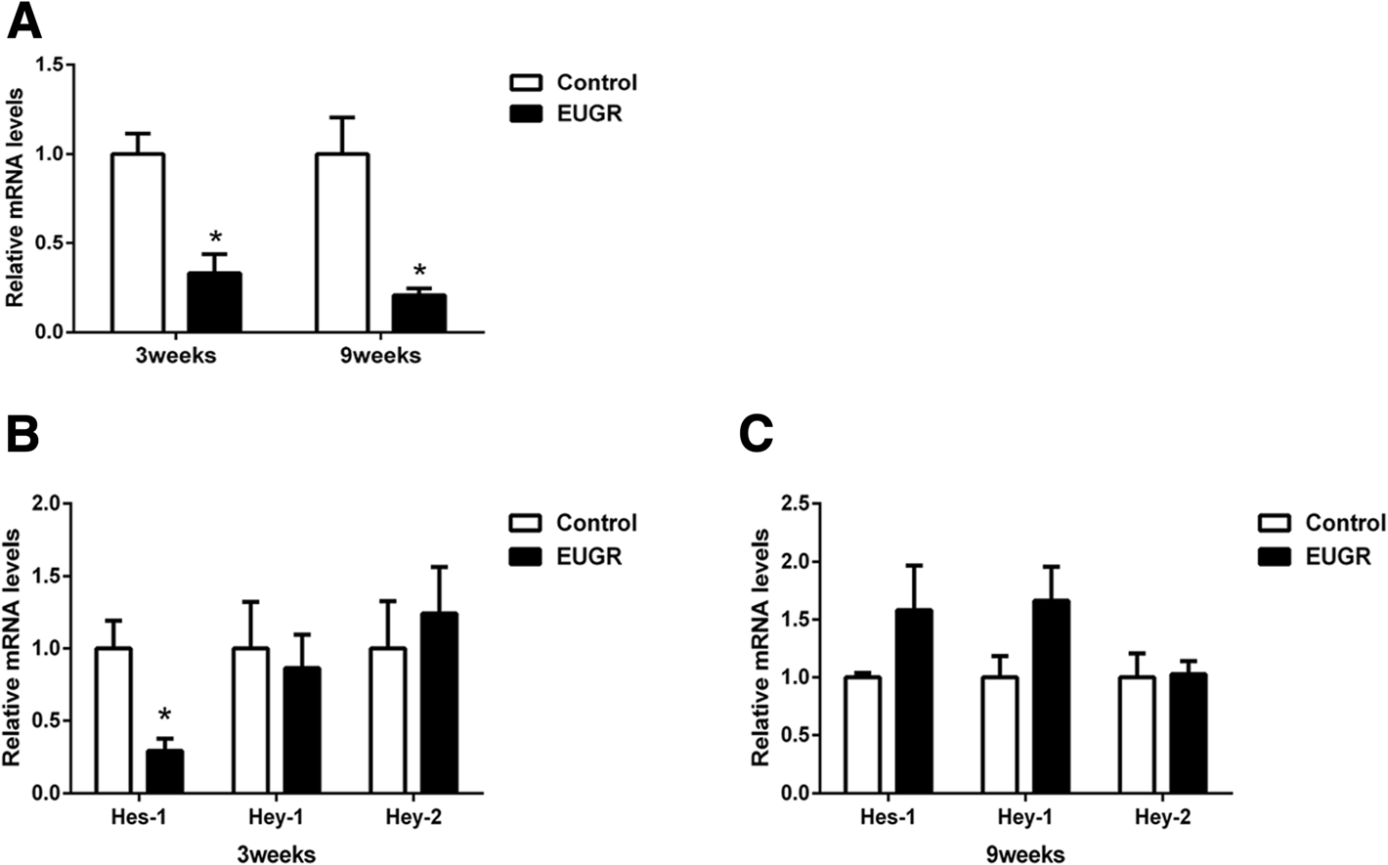
Expression levels of Notch1 gene (**a**), and ligands and downstream genes (**b** and **c**) in pulmonary vascular endothelial cells (PVECs) of control and EUGR male offspring rats at 3 and/or 9 weeks. Data are presented as mean ± SEM. (PVECs from 6–8 rats of each group were assessed). *P < 0.05

Notch1 mRNA levels at different time points were examined in PVECs. Compared with controls, Notch1 mRNA levels of EUGR rats were significantly decreased at 3 weeks (*P* = 0.031), and even lower at 9 weeks (*P* = 0.043, Fig. 3a). To determine whether Notch1 signaling was inhibited, the expression of Notch1 downstream targets was also measured. Of the three main downstream genes, *Hes-1* was decreased at 3 weeks, consistent with reduced Notch1 expression (Fig. 3b, *P* = 0.014), while there was no between-group difference at 9 weeks (Fig. 3c, *P* = 0.270).

### Effect of extrauterine growth restriction on histone modification of proximal Notch1 gene promoter

**Fig. 4:**
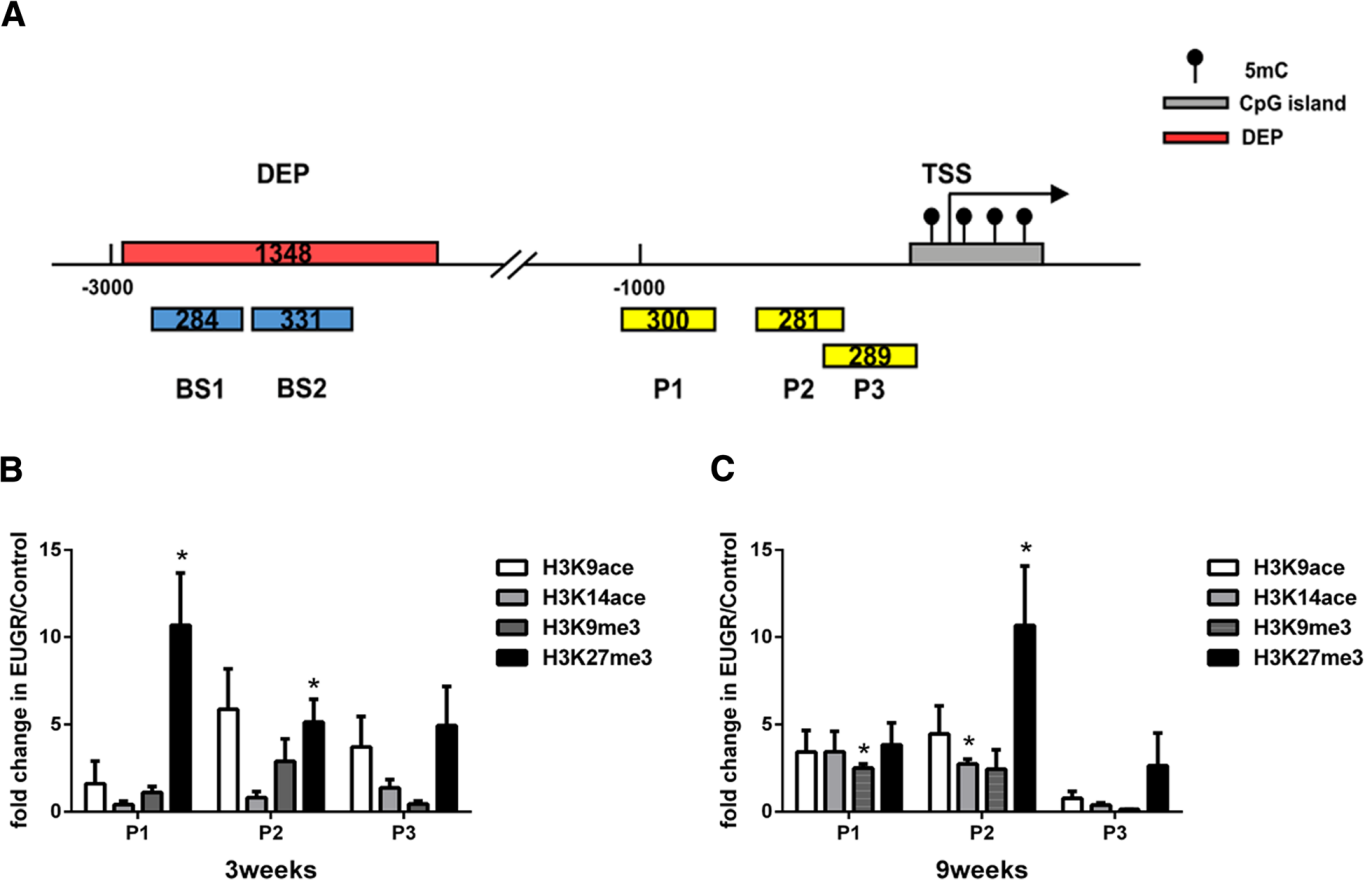
Histone modifications of the Notch1 gene promoter in rats at 3 and 9 weeks. **a** Schematic illustration showing the different enrichment peak (red bar) detected by Medip-chip in the distal promoter of the Notch1 gene and the region analyzed by bisulfite sequencing (BS) (blue bar). ChIP-qPCR analysis of regions proximal to the transcription start site are also indicated (yellow bar). The distribution pattern of four histone modifications at three sites in the proximal promoter (<−1500 bp) of Notch1 at (**b**) 3 weeks and (**c**) 9 weeks, analyzed by ChIP/real-time qPCR. Data are presented as mean ± SEM. (PVECs from 9 rats of each group were assessed). *P < 0.05

To investigate EUGR-induced Notch1 epigenetic changes, we analyzed DNA methylation and histone modification patterns in the Notch1 gene promoter region in PVECs. Combinations of histone modifications provide a measure of transcriptionally active (H3K9ace, H3K14ace) and repressed (H3K27me3) chromatin [24]. In this study, ChIP/real-time PCR was performed to examine the extent of four H3 covalent modifications (H3K9ace, H3K14ace, H3K9me3 and H3K27me3) at three regions in the proximal promoter (Fig. 4a). At 3 weeks, compared with controls, EUGR was associated with H3K27me3 at P1 (*P =* 0.033) and P2 (*P =* 0.037, Fig. 4b), whereas no difference was observed in other markers. In adult EUGR rats, a significantly increased H3K27me3 at P2 (>10 fold, *P =* 0.0498, Fig. 4c) was observed, in addition to increased H3K9me3 at P1 (*P =* 0.016) and increased H3K14ace at P2 (*P =* 0.044, Fig. 4c). Both young and adult EUGR rats exhibited significantly increased H3K27me3 at P1 and P2.

### Effect of extrauterine growth restriction on CpG methylation of Notch1 in the distal promoter

**Fig. 5:**
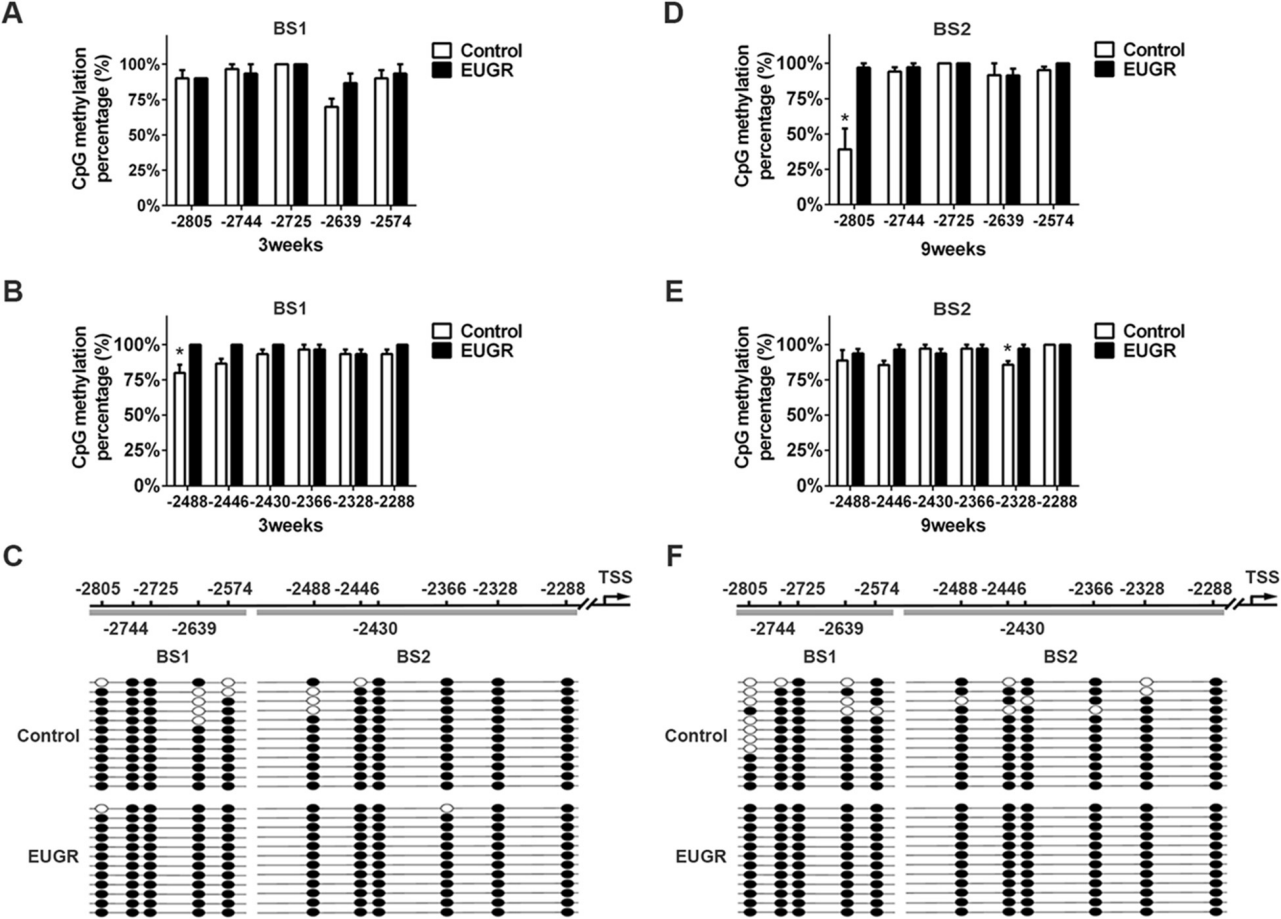
Altered DNA methylation levels of distal CpG sites in Notch1 promoter in postnatal and adult rats. CpG methylation profile in the BS1, BS2 of 3-week-old (**a**-**b**) and 9-week-old rats (**d**-**e**). White bars represent control values, black bars represent EUGR. Representative methylation sample are shown (**c**, **f**); each horizontal row of beads represents 11 CpG sites. Open circles indicate unmethylated CpG sites, while solid circles indicate methylated CpG sites. *P < 0.05. Data are presented as mean ± SEM. (*n* = 3 rats per group)

Cytosine methylation is a widespread DNA modification mechanism, which is influenced by the environment and affected by nutritional disturbance. Comprehensive analysis of DNA-methylation in PVECs was shown in a previous study by methyl-DNA immune precipitation chip (MeDIP-chip) using a genome promoter array [11]. A different enrichment peak exhibiting significant hypermethylation was detected at the distal promoter of the Notch1 gene (Fig. 5a). To address the degree of altered methylation of a single CG site, we assayed the methylation status of this region using BS in PVECs. Five CpG sites in BS1 and six in BS2 were analyzed within the different enrichment peak. At 3 weeks, EUGR increased CpG methylation at the −2488 CpG site (Fig. 5b, *P =* 0.026). At 9 weeks, the −2805 (Fig. 5b, *P =* 0.019) and −2328 CpG sites (Fig. 5e, *P =* 0.039) were significantly hypermethylated. Although no significant difference was found at other CpG sites, compared with control rats, more than half of these sites showed a slight trend toward hypermethylation, such as the −2639 and −2446 sites, in the EUGR rats, suggesting a general trend of hypermethylation of this locus at the distal promoter of the Notch1 gene. The effect may persist from early life into adulthood. The microarray data can be accessed from the Gene Expression Omnibus (GEO) site (http://www.ncbi.nlm.nih.gov/geo/) (accession number: GSE48648) [11].

**Fig. 6:**
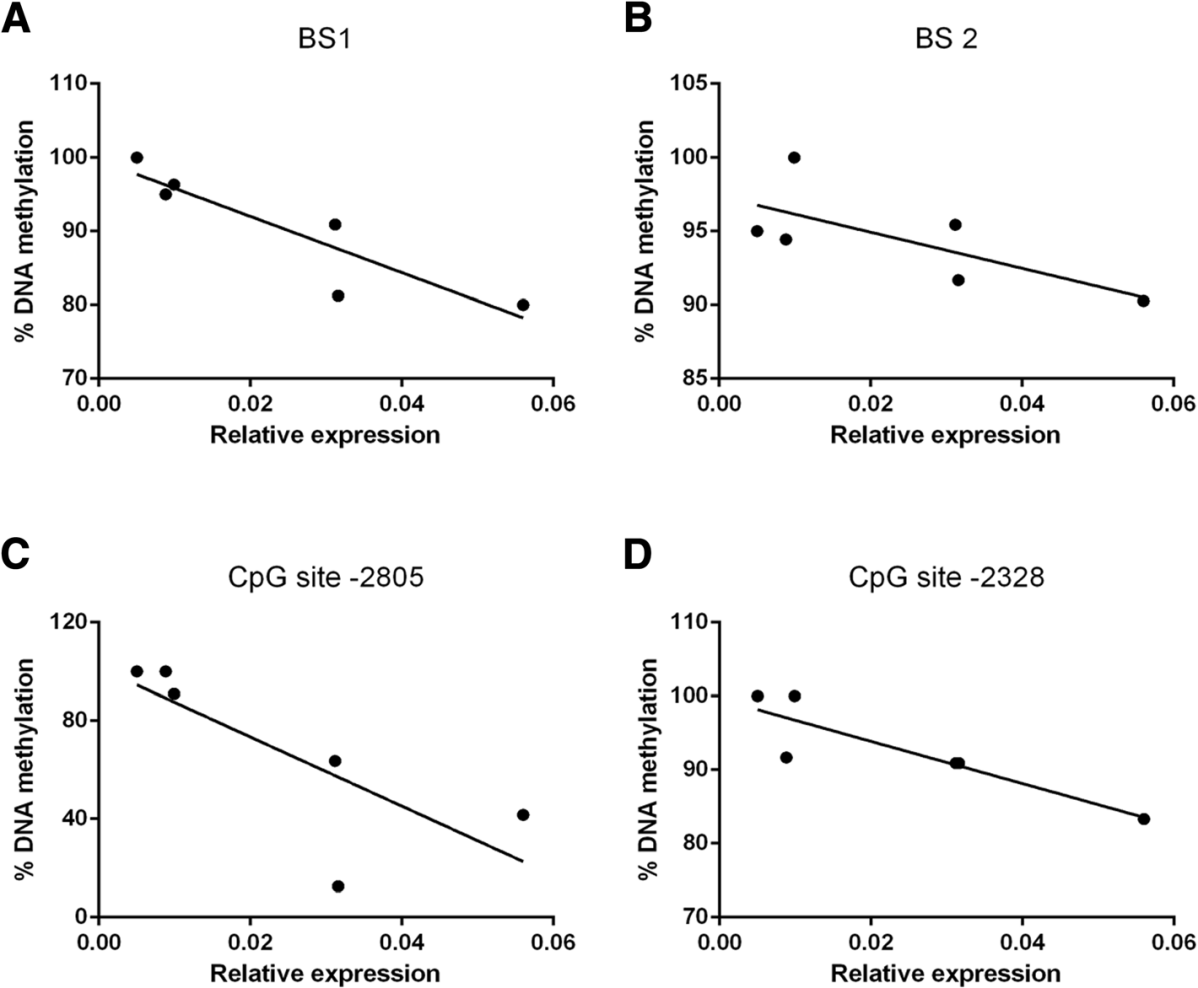
Correlations between Notch1 gene promoter DNA methylation and the level of mRNA expression at 9 weeks. Pearson correlation for relative gene expression and average DNA methylation of (**a**) BS1, (**b**) BS2. Correlation for relative gene expression and DNA methylation at (**c**) CpG site −2805, (**d**) CpG site −2328

We also attempted to correlate the degree of promoter methylation to the mRNA expression level of Notch1 and found that mRNA expression was inversely associated with methylation of BS1 (r^2^ = 0.82, *P* = 0.01) and CpG site −2328 (r^2^ = 0.79, *P* = 0.02) (Fig. 6a and d) at 9 weeks, while no significant correlations were observed between gene expression and methylation of BS2 (r^2^ = 0.51, *P* = 0.11) and the CpG site −2805 (r^2^ = 0.60, *P* = 0.07) (Fig. 6b and c). Despite slightly higher methylation at CpG site −2488 in EUGR at 3 weeks, no correlation was found (data not shown).

## Discussion

In our prior work, we documented EUGR-induced adult-onset pulmonary vascular dysfunction [11]. In the present study, we found that EUGR resulted in reduced pulmonary vascular density during early postnatal life. Decreased Notch1 expression in pulmonary endothelium was associated with reduced vascular density, which might be a consequence of altered epigenetic modification secondary to EUGR, including histone modification and DNA methylation at the Notch1 gene promoter.

Postnatal nutritional insult is a trigger event in the development of cardiovascular disease. Restriction of prenatal and early postnatal growth may increase systemic BP [33]. Our recent study demonstrated that EUGR male rats developed elevated PAP [11], which, for the first time, has been confirmed in the present study. However, no effect was observed on RVSP at 3 weeks, suggesting adult-onset pulmonary hypertension induced by EUGR. Although little attention has been paid in the literature concerning the negative impact of EUGR on cardiovascular function, a recent study [34] reported higher systolic and diastolic BP in prepubertal children with a history of EUGR, which supports our result.

The current study demonstrated decreased pulmonary vascular density in EUGR rats at 3 weeks, but not at 9 weeks as adults, suggesting an early postnatal disruption in pulmonary vasculature development, which was not yet sufficient to cause measureable hemodynamic changes in pulmonary circulation simultaneously. Rarefaction is considered as a predictor of adverse long-term cardiovascular prognosis [35–37]. Further recent studies demonstrated that impaired angiogenesis and peripheral muscle microcirculation rarefaction contribute to pulmonary arterial hypertension [16, 38]. Although neither of our study, nor other studies, observed microvascular rarefaction in adults [19], it has been reported in the early stages, or even prior to hypertension, in different organs [14, 39, 40]. Therefore, we postulate that microvascular rarefaction is a primary event in the development of nutritional programmed pulmonary hypertension.

Rarefaction of microvessels is a consequence of impaired angiogenesis, partly attributing to dysregulation of major factors in vascular development. As a key modulator, defects in endothelium Notch1 may result in impaired postnatal vascularization [21]. In the present study, we found significantly decreased Notch1 expression in PVECs in EUGR at 3 weeks compared with controls. Furthermore, consistently reduced expression was also observed in adult rats at 9 weeks. We detected several downstream genes of Notch1 simultaneously. Decreased *Hes-1* expression corresponded with decreased Notch1 levels at 3 weeks, indicating Notch1-mediated downregulation of *Hes-1* following EUGR. Our results show EUGR-related decreased Notch1 caused inhibition of *Hes-1*. However, further studies are needed to elucidate how the dysfunction of Notch1 signaling contributes to the disruption of pulmonary vascular development.

Epigenetic adaptation, including histone modification and DNA methylation, is an important molecular mechanism underlying perinatal insult-related cardio-metabolic disorder [41, 42]. Histone acetylation often associates with gene activation, including H3 acetylation at K9 and K14, whereas histone trimethylation at K9 and K27 is correlated with gene silencing [43, 44], known as repressive histone mark. In our current study, although H3K9me3 and H3K14ace were increased in EUGR adult rats, H3K27me3 was more significantly increased at both 3 and 9 weeks in PVECs. In association with the consistently reduced Notch1 expression, our data suggest that repressive histone mark (H3K27me3) plays a dominant role in Notch1 gene expression. This is supported by a report of significantly higher H3K27me3 in patients with systemic lupus erythematosus [45]. Additionally, the persistent trend of increased H3K27me3 at 3 and 9 weeks in the present study implies a possible epigenetic adaptation induced by EUGR.

Abnormal adaptation of DNA methylation during development can persist into adulthood [46, 47]. Although CpG islands at gene promoters are the most studied regions of DNA methylation, isolated CpGs in the distal promoter can also have regulatory significance [48]. Previously, MeDIP, and genome-wide microarrays (promoter and CpG islands) were employed to identify a genome methylation pattern in EUGR and control rats. A significant hypermethylation peak was assessed in the distal Notch1 gene promoter in PVECs of EUGR adult rats [11]. In the current study, BS was performed to determine the degree of altered methylation of single CpG sites in this region. We observed two CpG sites with significantly increased methylation in EUGR rats, such as the −2488 CpG site at 3 weeks, and the −2805 and −2328 CpG sites at 9 weeks. Considering the significantly increased H3K27me3 in our study, epigenetic down-regulation of Notch1 seems to be mediated by a combination of DNA methylation and histone modification [45]. Although the relatively modest increase of Notch1 DNA methylation in EUGR is not surprising, a significantly inverse correlation was demonstrated between the degree of methylation of BS1, CpG site −2328 and Notch1 gene expression. Therefore, our result gives new insights into the environmental stress-sensitive regulating region of the Notch1 gene in the distal promoter through altered DNA methylation.

Finally, the limitation of this study should be addressed. Although we have now provided evidence that EUGR-induced epigenetic down-regulation of Notch1 may contribute to pulmonary vascular rarefaction, the mechanism underlying these altered epigenetic modification is still unclear. The epigenetic pattern is usually governed by specialized enzymes, including histone deacetylase, histone methytransferases and DNA methyltransferases. It has been demonstrated that abnormal Notch1 gene expression involves altered recruitment of these enzymes, such as enhancer of zeste homolog 2 (EZH2) and DNA methyltransferase (DNMT-3b), to promoter regions and/or additional cis-regulatory regions in the Notch1 gene [30]. Therefore, future studies need to elucidate the underlying mechanism.

## Conclusion

Our findings indicate that EUGR-induced decreased pulmonary vascular density in early postnatal life correlates with decreased Notch1 gene expression in endothelial cells. Furthermore, we documented that a combination of epigenetic adaptations, including increased H3K27me3 in the proximal promoter and hypermethylation of CpG sites in the distal promoter, were associated with decreased Notch1 expression and its target gene, *Hes-1*. More importantly, we provide new evidence that pulmonary vascular rarefaction is an important predictive event in the early nutritional programming of elevated PAP.

## Additional file

Additional file 1: Table S1.
**Primers used for the amplification of genes.**

## Abbreviations

EUGR: Extrauterine growth restriction
PVEC: Pulmonary vascular endothelial cell
RVSP: Right ventricular systolic pressure
PAP: Pulmonary arterial pressure
IUGR: Intrauterine growth restriction
BP: Blood pressure
MACS: Magnetic-activated cell sorting
ChIP: Chromatin immunoprecipitation
BS: Bisulfite sequencing
MeDIP-chip: Methyl-DNA immune precipitation chip
PBS: Phosphate-buffered saline
Hes-1: Transcription factor hairy and enhancer of split-1
