# Supplementary material for: Epigenetics of Notch1 regulation in pulmonary microvascular rarefaction following extrauterine growth restriction
Source: Respir Res. 2015 Jun 4;16(1):66. doi: 10.1186/s12931-015-0226-2 (PMC4486133; doi:10.1186/s12931-015-0226-2)
Supplement: Additional file 1: Table S1. — Primers used for the amplification of genes. [file 12931_2015_226_MOESM1_ESM.pdf]

Addtional file 1:Table S1. Primers used for the amplification of genes

| Gene | | Forward | Reverse |
|---|---|---|---|
| **For RT-qPCR** | | | |
| β-actin | | GCCAACCGTGAAAAGATG | TGCCAGTGGTACGACCAG |
| Notch1 | | CACCCATGACCACTACCCAGTT | CCTCGGACCAATCAGAGATGTT |
| Hes-1 | | CAACACGACACCGGACAAAC | CGGAGGTGCTTCACTGTCAT |
| Hey-1 | | TGGCTGAAGTTGCCCGTTAT | TGTGTGGGTGATGTCCGAAG |
| Hey-2 | | GCTTCCGTACCTCTGGTGAG | TCCCGTTCGTGTGAGTGAAC |
| **For ChIP-qPCR analysis** | | | |
| P1 | | GGGAGGCTCGTAGGACAA | CGTTCCACGGTAACTCTTC |
| P2 | | TTTAAGGTCAAGCGTTCCG | GCATTTCAATAGTAGAGGAGGT |
| P3 | | GGTGCAGCTACGCCTTTC | CCCTTGTGGACCCTGTCTC |
| **For Bisulphite Sequencing(nested PCR)** | | | |
| BS1 | inner | TTTTTGTGATTGGTGTTGGTTATT | ACCCCTTCTAAAACTTCCTAAACAT |
| | outer | TTTAAGTATATTTTAGGGTTTTTGTTT | CTAAAAAACTAATCAATACATTACC |
| BS2 | inner | ATTAGTTGTTTTGGAGGGAGTAGGT | AAAAACCCAAACAAACAAAATATCA |
| | outer | TTTAAGGAGAGGTTTAGAAGTATTTTTAGGAGA | CTAAAATAACCAACACCAATCACAA |
